# Nutrigenetic Investigations in Preeclampsia

**DOI:** 10.3390/nu16193248

**Published:** 2024-09-26

**Authors:** Zoltán Kukor

**Affiliations:** Institute of Biochemistry and Molecular Biology, Semmelweis University, 1094 Budapest, Hungary; kukor.zoltan@semmelweis.hu

**Keywords:** preeclampsia, nutrient, polymorphism, BMI, MTHFR, VDR

## Abstract

**Background**: Preeclampsia is a leading cause of pregnancy-related maternal and fetal morbidity and mortality. Although its precise cause and prevention remain unclear, risk factors such as overweight and inadequate nutrient intake (e.g., calcium, folic acid, and vitamin D) are known to increase its incidence. Recent research has focused on the genetic predisposition to preeclampsia, identifying polymorphisms that may affect enzyme or receptor function. This study aims to review existing literature examining the relationship between genetic polymorphisms, BMI (body mass index), and nutrient levels in preeclampsia to develop more actionable therapeutic strategies. **Methods**: A systematic review was conducted to analyze studies on the nutrigenetic relationship between BMI, micronutrients, and preeclampsia. **Results**: A total of 17 studies investigating 12 genes related to BMI and 10 studies exploring 3 genes in relation to micronutrient levels were included in the analysis. Several polymorphisms associated with preeclampsia were found to be influenced by maternal BMI or serum vitamin levels. The interactions between certain gene variants and these factors suggest that both BMI and micronutrient status may modify the risk of developing preeclampsia in genetically predisposed individuals. **Conclusions**: Our findings emphasize the potential for reanalyzing existing data by categorizing based on genotype and nutrient levels. This approach could yield more personalized dietary and therapeutic recommendations for managing preeclampsia. In the future, genetic information may support the development of tailored nutritional counseling during pregnancy to mitigate preeclampsia risk.

## 1. Introduction

Preeclampsia is a pregnancy-specific disease that occurs in 2–8% of pregnancies. It is one of the leading causes of maternal and fetal mortality related to pregnancy. The causal treatment is not yet known [1,2]. Developing effective prevention methods is an important goal. The traditional definition of preeclampsia is based on the development of gestational hypertension and proteinuria. According to the 2018 recommendations of the International Society for the Study of Hypertension in Pregnancy (ISSHP), proteinuria is **not** required for a diagnosis of preeclampsia. Preeclampsia is gestational hypertension (systolic blood pressure ≥140 and/or diastolic blood pressure ≥90 mmHg after 20 weeks of gestation) accompanied by one or more of the following new-onset conditions at or after 20 weeks of gestation: (1) proteinuria, (2) other maternal organ dysfunction, including acute kidney injury, liver involvement, neurological complications, and hematological complications, and (3) uteroplacental dysfunction [3]. The American College of Obstetrics and Gynecology (ACOG) definition of preeclampsia was based on the development of at least one of the following: new-onset proteinuria, renal insufficiency in the absence of underlying renal disease, hepatic involvement with serum transaminases more than twice the upper limit of normal, thrombocytopenia, neurologic complications, or pulmonary edema [4]. Compared with the traditional and ACOG definitions of preeclampsia, the International Society for the Study of Hypertension in Pregnancy maternal–fetal factors plus angiogenic imbalance best identified the adverse outcomes. Compared with the traditional and ACOG definitions of preeclampsia, the more inclusive ISSHP definition of maternal end-organ dysfunction seems to be more sensitive. The addition of uteroplacental dysfunction to the broad definition optimizes the identification of women and babies at risk, particularly when angiogenic factors are included [5].

The likelihood of developing preeclampsia increases if there are close relatives who have had the disease [6], suggesting a potential hereditary component, which has been considered for a long time. Many polymorphisms associated with preeclampsia have been studied. The results are contradictory, as several factors influence the effects of these polymorphisms, including race. For multiple gene polymorphisms, increased risk of preeclampsia has been observed only in certain populations [7,8,9,10,11,12].

In a recently published review, Esquivel summarized the nutrients that may aid in the prevention of preeclampsia. Strong evidence supports that consuming 25–30 g of fiber daily reduces the risk of preeclampsia. From the 20th week of pregnancy, a daily 1000 mg Ca supplementation is recommended, regardless of dietary calcium intake. Additionally, 400–1000 IU/day of vitamin D also has a protective role against preeclampsia. Milk-based probiotics have been shown to have beneficial effects as well. Some nutrients and supplements, although hypothesized to have protective roles, have not been supported by evidence, with studies yielding contradictory results. These include vitamins A and C, zinc (for oxidative stress reduction), magnesium (due to its blood-pressure-lowering effects), salt intake restriction (also for blood pressure control), and Omega-3 fatty acids (with antihypertensive, antioxidant, and anti-inflammatory effects) [13].

Overweight increases the risk of several pregnancy-associated diseases, including gestational hypertension, gestational diabetes mellitus (GDM), and preeclampsia [14]. Increased adipose tissue itself contributes to the development of preeclampsia. Preeclampsia is associated with increased concentrations of adipocyte-derived metabolites, such as free fatty acids [15,16], TNF-α, IL-6 [17,18], leptin, adiponectin [19], and others [20,21].

The aim of this study is to review the existing nutrigenetic research related to preeclampsia. Specifically, it aims to explore whether the effects of polymorphisms that increase or decrease the risk of preeclampsia are influenced by the concentration of dietary components interacting with the gene product (protein), such as cofactors or transported ligands. Overweight is known to increase the incidence of preeclampsia. Therefore, it is important to highlight polymorphisms associated with preeclampsia, whose effects may be modified by BMI, either increasing or potentially decreasing the risk of preeclampsia.

## 2. Obesity and Preeclampsia

Nutrition also influences the incidence of preeclampsia. Pre-pregnancy overweight (BMI > 30 kg/m^2^) increases the risk by 2–3 times. Preeclampsia is significantly more prevalent in women with overweight and obesity. On the other hand, preeclampsia is significantly lower in women classified as underweight [2,14,22]. Obesity is the only risk factor with a ‘definite’ association with preeclampsia based on high-quality evidence [23]. As the number of overweight individuals continues to rise, this could also increase the incidence of preeclampsia. The WHO defines overweight as a BMI equal to or more than 25, and obesity as a BMI equal to or more than 30. In 2016, more than 1.9 billion adults were overweight and 650 million were obese [24]. Prevalence of obesity increased from 6.4% in 1975 to 14.9% in 2014 in women [25]. The risk of developing preeclampsia showed a nearly two-fold rise among overweight mothers, a three-fold rise in obese mothers, and a significantly lower risk in underweight mothers [22].

### 2.1. Method of Searching

Data (until publication on 1 July 2024) were collected from the PubMed, Web of Science, and Scopus databases. I used the keywords “BMI” OR “body mass index” AND “preeclampsia” AND “polymorphism” as I conducted my study.

Inclusion criteria were human investigations with genotype determination and differential values of BMI. Exclusion criteria were non-human studies, reviews, and in vitro studies.

**Figure 1:**
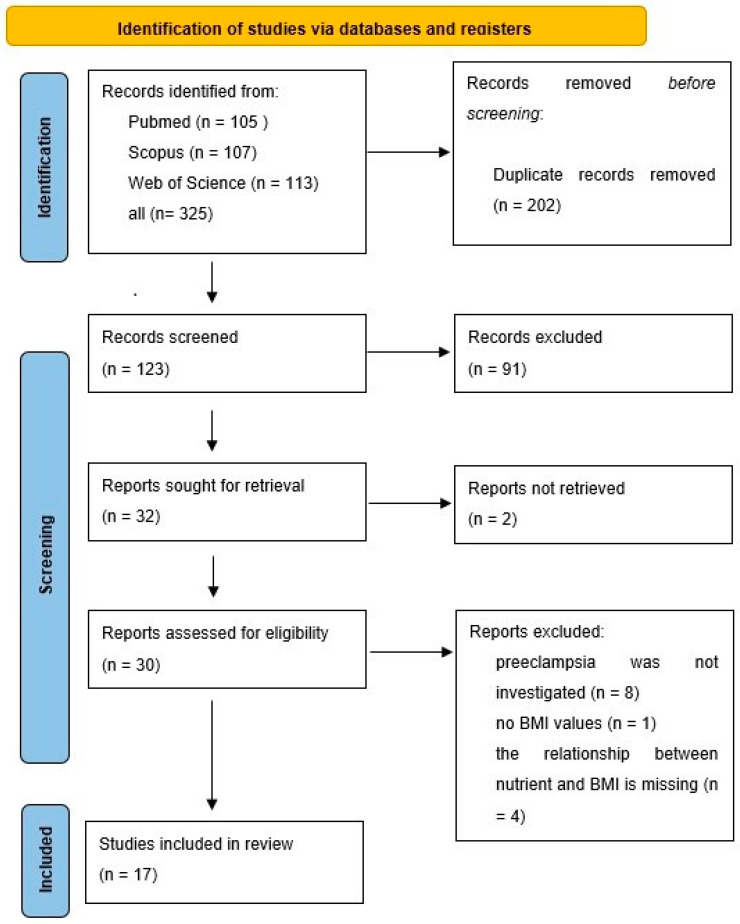
Flow diagram of the search for BMI studies.

The PRISMA flow diagram of the search is shown in Figure 1.

During the search, polymorphisms of 12 genes were found, which were also associated with BMI data.

### 2.2. REN (Renin Gene)

Socol et al. investigated the renin rs5707 polymorphism in a Romanian normotensive control group (n = 254) and preeclamptic patients (n = 146) [26]. Preeclampsia was not defined. The renin rs5707 (C4280A [27]) polymorphism is located at the 4th intron [28]. The AA genotype, associated with a reduced risk of developing preeclampsia, was significantly more frequent in women in the control group with a BMI < 24 kg/m^2^. The AA genotype was considerably less common in women in the preeclamptic/eclamptic group with a BMI ≥ 24 kg/m^2^. The study results indicated that overweight counteracted the beneficial protective effect of the genotype against preeclampsia [25].

Yu et al. investigated renin rs5707 and rs5705 polymorphisms in 347 preeclamptic and 700 healthy control pregnant Chinese participants. Preeclampsia was defined according to the 2022 ACOG [29]. After Bonferroni correction, no association with preeclampsia was found for the rs5705 polymorphism. The renin rs5707 AC genotype in combination with a pre-pregnancy BMI ≥ 24 kg/m^2^ was significantly associated with an increased risk of preeclampsia and eclampsia [30].

### 2.3. AGT (Angiotensinogen Gene) and AGTR1/2 (Angiotensin II Receptor Type 1 and 2 Genes)

He et al. examined Chinese participants, including 228 preeclamptic and 358 controls. Angiotensinogen rs7079 polymorphism was investigated. Preeclampsia was diagnosed by diagnostic criteria of the ACOG [31]. The rs7079 (C11525A) polymorphism is on the angiotensinogen gene 3′UTR side. The polymorphism is in the binding site of miR-31 and miR-584 [32]. The rs7079 T allele was significantly related to the decrease of *AGT* expression levels [20]. The rs7079 TT genotype significantly increased preeclampsia risk in the subgroup with BMI < 25.

Zhou et al. investigated polymorphisms of *AGTR1* (angiotensin II receptor type 1 A1166C) and *AGTR2* (C4599A, A1675G, and T1134C) as a function of BMI in preeclamptic (n = 123) and uncomplicated pregnancies (n = 1185). Caucasian parent–infant trios were included in the study. Preeclampsia was defined as systolic blood pressure ≥140 mmHg and/or diastolic blood pressure ≥90 mmHg after 20 weeks of gestation with proteinuria (24 h urinary protein ≥300 mg/24 h, or spot urine protein:creatinine ratio ≥30 mg/mmol creatinine, or urine dipstick protein ≥++) or any multisystem complication of preeclampsia. Significant associations were observed for *AGTR2* C4599A. The *AGTR2* C4599A polymorphism in mothers, fathers, and babies was linked to preeclampsia, but this association was evident only in pregnancies where the women had a BMI ≥ 25 kg/m^2^, indicating a gene–environment interaction [33].

### 2.4. ACE2 (Angiotensin-Converting Enzyme 2 Gene)

Huang et al. studied the polymorphisms of *ACE2* (angiotensin-converting enzyme 2) involving Chinese participants (n = 327 with preeclampsia and n = 591 controls). The criteria for preeclampsia were as follows: first presentation after 20 weeks of gestation with systolic blood pressure ≥140 mmHg or diastolic blood pressure ≥90 mmHg and urine protein ≥0.3 g/24 h. The rs2106809 A > G is an intron variant [34]. Among patients with BMI > 23 kg/m^2^, a significant correlation was observed between rs2106809 A > G and preeclampsia risk. The AA genotype significantly increases the risk of preeclampsia if the pre-pregnancy BMI is above 23 kg/m^2^ [35].

### 2.5. RGS2 (Regulator of G-Protein Signaling 2 Gene)

Karppanen et al. investigated the relationship between the regulator of G-protein signaling 2 gene (*RGS2*) rs4606 polymorphism and preeclampsia. *RGS* proteins inhibit signal transduction by stimulating GTP dissociation from the alpha subunit of heterodimeric G proteins. *RGS2* inhibits vasoconstrictor signaling pathways, thereby reducing blood pressure. The rs4606 variant is associated with low RGS2 levels, which may contribute to hypertension. In this study, preeclamptic (n = 1339) and healthy control (n = 697) groups were compared. The patient group included preeclamptic women (systolic blood pressure ≥140 mmHg or diastolic blood pressure ≥90 mmHg, proteinuria ≥0.3 g/24 h, or ≥1+ dipstick) and those with superimposed preeclampsia (elevated blood pressure predating mid-pregnancy, including both women with chronic hypertension and those with de novo hypertension before mid-pregnancy). No association was found when comparing all preeclamptic and control participants. However, an intriguing association emerged during subgroup analysis by BMI. The CG/GG genotypes increased the risk of preeclampsia in the BMI 25 ≤ BMI < 30 kg/m^2^ subgroup. This association was not significant in groups with BMI below 25 or above 30 [36].

### 2.6. VDR (Vitamin D Receptor Gene)

Ghorbani et al. investigated the vitamin D receptor (*VDR*) rs7975232 polymorphism in a Kurdish population (n = 100 preeclamptic and n = 100 control). The preeclampsia was defined as systolic blood pressure ≥140 mmHg, diastolic blood pressure ≥90 mmHg, the excretion of proteinuria >300 mg protein/24 h, a urine protein:creatinine ratio of >0.3, and ≥30 mg/dL protein in a random urine sample (1+ reaction on a standard urine dipstick). GT genotype was associated with the 2.55-fold increased risk of preeclampsia. The GT + TT genotype of *VDR* compared to the GG genotype was associated with a significantly higher level of BMI and systolic blood pressure. The *VDR* rs7975232 polymorphism was associated with higher BMI and systolic blood pressure and lower vitamin D levels and was also associated with preeclampsia. Mean BMI was 20 kg/m^2^ with the GG genotype, compared to 25.4 kg/m^2^ in the presence of the GT + TT genotype. Also, in subjects that were carriers of the GT + TT genotype, the systolic blood pressure was significantly higher (135.5 ± 23.8 mmHg, *p* = 0.026), compared to those with the GG genotype (124.8 ± 19 mmHg) [37].

### 2.7. AVP (Arginine Vasopressin Gene)

Erfanian et al. studied three polymorphisms of arginine vasopressin (*AVP*; rs3729965, rs3761249, and rs61138008) in an Iranian population (n = 100 preeclamptic and n = 100 control). Preeclampsia was defined as high blood pressure (systolic/diastolic blood pressure ≥140/90 mmHg) and proteinuria (≥+1 on dipstick). Obesity was defined as BMI ≥ 30 kg/m^2^. A connection with preeclampsia was observed only for the rs3729965 polymorphism, and only when BMI was also taken into account. Their analysis showed that the T allele was correlated with high BMI in the preeclamptic group [38].

### 2.8. ACVR2 (Activin A Receptor Type 2A Gene)

In their 2019 publication, Amosco et al. described the relationship between the *ACVR2A* gene (Activin A Receptor Type 2A) rs1014064 polymorphism and BMI. The study included 150 preeclamptic and 175 uncomplicated normal Filipino pregnancies. Preeclampsia was defined as systolic blood pressure ≥140 mmHg and/or diastolic blood pressure ≥90 mmHg and proteinuria (≥300 mg protein/24 h or a urine protein dipstick of ≥2+) after 20 weeks of gestation. Their data suggested that a BMI above 25.1 kg/m^2^ increases the risk of preeclampsia in carriers of the rs1014064 G allele [39].

### 2.9. LGALS13 (Placental Protein 13 Gene)

Madar-Shapiro et al. investigated the polymorphisms of the LGALS13 gene (placental protein 13) in a mixed ethnic population, including preeclamptic (n = 70) and unaffected control groups (n = 200). The definition of preeclampsia was in accordance with the ISSHP. The AA genotype of the rs3764843 polymorphism increased the risk of preeclampsia. In the obese group (BMI ≥ 35), the risk significantly increased. The study also demonstrated that the expression of placental protein 13 was reduced in BeWo cells with the AA genotype [40].

Zhang et al. investigated the *beta3-adrenergic receptor gene* rs4994 A/G polymorphism in non-Hispanic Caucasian participants, including preeclamptic (n = 87) and normotensive (n = 213) pregnant women. Preeclampsia was defined as sustained pregnancy-induced hypertension (systolic/diastolic blood pressure ≥140/90 mmHg and/or a sustained 15 mmHg diastolic rise or a 30 mmHg rise in systolic blood pressure above first trimester blood pressure values) with proteinuria (urine protein concentrations ≥30 mg/dL or ≥1 + on a urine dipstick) [41]. The Trp64Arg mutation (AG) was associated with a ten-fold decrease in adipocyte receptor sensitivity [42].

### 2.10. miR-27a

Maharaj et al. studied HIV-infected Black South African women with preeclampsia (n = 98) and normotensive pregnancies (n = 95). Preeclampsia was defined as blood pressure ≥140 mmHg systolic or ≥90 mmHg diastolic after 20 weeks of gestation and proteinuria (≥+1 on urine dipstick testing). They analyzed the *miR-27a* rs895819 polymorphism but found no association between allele distribution and preeclampsia. They also analyzed the relationship between BMI and allele distribution. In the normotensive control group, the BMI of individuals with the TT genotype (34.3 ± 2.3 kg/m^2^) was significantly higher than those with the CT/CC genotypes (29.0 ± 1.0 kg/m^2^). Interestingly, in the preeclamptic group, the relationship was reversed but not significant (TT vs. TC/CC: 29.1 ± 1.1 vs. 32.6 ± 1.3 kg/m^2^ BMI). When examining HIV-infected and non-infected subgroups, a significant difference in BMI by genotype was found only in the preeclamptic HIV-infected group (TT vs. TC/CC: 27.9 ± 0.7 vs. 33.5 ± 1.7 kg/m^2^ BMI). It is important to note that, unlike other studies, these BMI values were measured at the first hospitalization, not pre-pregnancy [43].

### 2.11. LIPC (Hepatic Lipase Gene)

Lin et al. investigated polymorphisms in the promoter region of the human hepatic lipase (*LIPC*) gene in healthy normotensive (n = 331) and gestational hypertensive (n = 321) pregnant groups. Within the hypertensive group, they also examined preeclampsia, separating it into severe (n = 207) and mild preeclampsia (n = 56) subgroups. Unfortunately, data for the severe preeclampsia group are missing from the publication. The mild preeclampsia was defined as blood pressure (BP) 160/110 ≥ BP ≥ 140/90 mmHg and proteinuria between 300 and 500 mg/24 h urine, or +1–2 on a dipstick. The important result from our perspective is that for the *LIPC* −250G/A (rs2070895) polymorphism, in the mild preeclampsia group, the BMI of individuals with the AA genotype (34.2 ± 1.8) was significantly higher than that of the GA/GG group (28.2 ± 0.7). No significant differences in BMI were observed for the other polymorphism (rs1800588) or in the gestational hypertensive group. Similar data for the control group are unfortunately missing [44].

Enquobahrie et al. also studied hepatic lipase. They investigated the relationship between the rs1800588 (−514 C/T) polymorphism and pre-pregnancy overweight in preeclamptic (n = 157) and normotensive control (n = 180) pregnant women from Peru. Preeclampsia was defined according to the ACOG criteria. Women with overweight and the *LIPC* TT genotype had a significant 3-fold increased risk of preeclampsia, as compared to not overweight and the CC/CT genotype [45].

### 2.12. MIF (Macrophage Migration Inhibitory Factors Gene)

I only had access to the abstract of the publication by Li et al. The study examined the macrophage migration inhibitory factor −173G/C polymorphism in severe preeclamptic (n = 124) and healthy control (n = 160) groups. No differences were found in genotype distribution, but severe preeclampsia patients with CG/CC genotypes had higher BMI values (25 ± 4 kg/m^2^) compared to those with the GG genotype (22 ± 4 kg/m^2^). The CG/CC genotypes also increased the degree of insulin resistance [46].

### 2.13. GWAS Studies

Ardissino et al. used inverse-variance-weighted Mendelian randomization to assess the association between BMI and all gestational outcomes (n = 136,325) of the (pre)eclamptic group (n = 4743). The study was conducted in a European population. Due to the use of multiple studies in the analysis, preeclampsia could not be defined. Higher genetically predicted BMI was associated with an increased risk of preeclampsia [47].

Abramova et al. conducted a GWAS study on pregnant Russian women (control n = 498 and preeclampsia n = 452). The participants were divided into two groups: BMI < 25 (control n = 339 and preeclampsia n = 290) and BMI ≥ 25 (control n = 159 and preeclampsia n = 162). Participants were classified into the preeclampsia group based on the ACOG criteria. The following BMI-specific associations with preeclampsia were found: rs805303 of *BAG6* (BCL-2-associated athanogene6 [48]; protective allele: A) and rs167479 of RGL3 (activated Ras-binding protein [49]; risk allele: G), associated with preeclampsia in women with a BMI ≥ 25 and not associated with the disorder in the BMI < 25 group [50].

Summarizing the genotypes associated with BMI, Table 1 shows the results. Studies missing from the table are those in which the effect of the genotype on the risk of preeclampsia in obese individuals was not examined [30,40,41], or where the specific genotype data were missing [31].

## 3. Micronutrients and Preeclampsia

Nutrient deficiencies increase the risk of preeclampsia. Supplementing these nutrients reduces the incidence of preeclampsia [41,51]. The most well-known among these are Ca^2+^ [52,53], folic acid [54,55], and vitamin D [56,57,58].

### 3.1. Method of Searching

Data (until publication on 1 July 2024) were collected from the PubMed, Web of Science, and Scopus databases. I used the keywords “nutrients” OR “micronutrients” AND “preeclampsia” AND “polymorphism” as I conducted my study. A new search was performed with the genes investigated in the publications obtained as a result of the nutrient or micronutrient search. I selected the publications I found. The publication found in this way was also included in the review.

Inclusion criteria were human investigations with genotype determination. Exclusion criteria were non-human studies, reviews, and in vitro studies.

**Figure 2:**
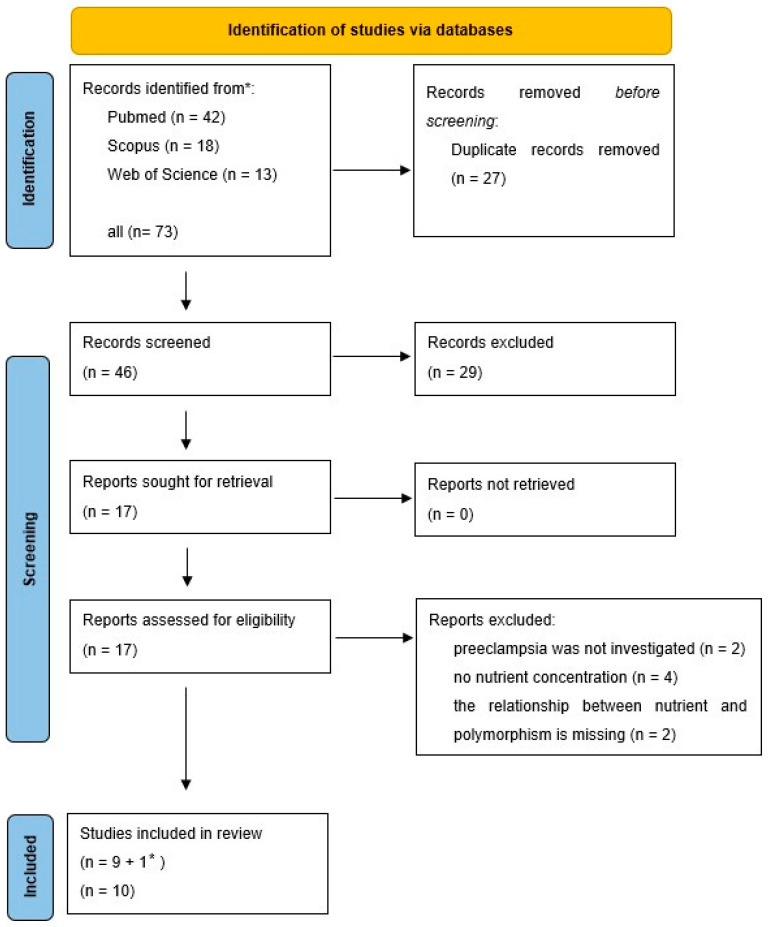
Flow diagram of the search for nutrients/micronutrients studies. * A new search was performed with the genes investigated in the publications obtained as a result of the nutrient or micronutrient search. I selected the publications I found. The publication found in this way was also included in the review.

The PRISMA flow diagram of the search is shown in Figure 2.

During the search, polymorphisms of 3 genes were found, which were also associated with micronutrients data.

### 3.2. Methylenetetrahydrofolate Reductase (MTHFR) and Folic Acid

The one-carbon units attached to folic acid are essential in DNA synthesis and repair and as a source of methyl groups in methylation reactions. This includes DNA and histone methylation, so folic acid also plays a role in epigenetic regulation. *MTHFR* catalyzes the conversion of 5,10-methylenetetrahydrofolate to 5-methyltetrahydrofolate. This enzyme, therefore, channels one-carbon units away from purine and pyrimidine synthesis and into the provision of methyl groups for S-adenosylmethionine-mediated methylation reactions [59]. Two common polymorphisms of the human enzyme have been identified: C677T (Ala222Val) and A1298C (Glu429Ala). The Glu429Ala protein has biochemical properties that are indistinguishable from the wild-type enzyme. The Ala222Val *MTHFR*, however, has an enhanced propensity to dissociate into monomers and to lose its FAD cofactor on dilution. The resulting loss of activity is slowed in the presence of methyltetrahydrofolate or adenosylmethionine [60,61].

Mislanova et al. investigated the *MTHFR* C677T polymorphisms in pregnant Ukrainian women, comparing those without gestational complications (n = 40) and those with preeclampsia (defined as diastolic blood pressure >90 mmHg and proteinuria; n = 28). The wild-type CC (Ala/Ala) enzyme activity was considered 100%, with CT activity at 66% and TT (Val/Val) activity at only 30%. They measured folate concentrations in the placentas, as well as homocysteine and methionine levels. Clinical data were not included in the publication. The prevalence of *MTHFR* genotypes was not significantly different between the control and preeclamptic groups. Placental folate content dependent on genotype was measured. The folate content was significantly lower in the samples from preeclamptic pregnancies with a C/T genotype and higher in the samples with a T/T genotype. The samples from other categories revealed no statistically significant differences [62].

Williams et al. investigated the C677T polymorphism and folate levels in preeclampsia within a Peruvian population. Maternal serum folate concentrations were measured at 36–37 weeks of gestation in preeclamptic (n = 125) and normotensive control (n = 179) participants. Preeclampsia was defined as a persistent rise of 15 mmHg in diastolic blood pressure, a 30 mmHg rise in systolic blood pressure, or a persistent blood pressure of at least 140/90 mmHg and urine protein concentration of 30 mg/dL or more (or 1 + on a urine dipstick) [63].

Clinical data were also not included in this publication. No significant differences were found between the two groups either in genotype distribution (CT or TT: 68.8% in preeclamptic women and 64.3% in the control group) or in low maternal serum folate levels (<9.2 nM: 35% in preeclamptic women and 24% in the control group). However, when the analysis was performed by combining the two genotype groups (CC and TC/TT) and categorizing based on folate levels, the following results were observed:

- For the CC genotype (n = 97), the incidence of low folate levels was similar in both preeclamptic and control cases (10% in each group). However, in the normal folate range, the percentage of preeclamptic cases (28%) was significantly lower compared to the control group (52%).
- For the TC or TT genotypes (n = 200), similar results were found. The incidence of preeclampsia with low folate levels was 17% in both preeclamptic and control groups, whereas with normal folate levels, preeclamptic cases were 26%, compared to 40% in the control group.

This suggests that adequate folate levels have a protective effect, while the protective effect of the CC genotype against preeclampsia is less pronounced [63].

Powers et al. studied the *MTHFR* C677T homozygous genotypes, including 15 CC and 15 TT controls, and 14 CC and 13 TT cases of preeclampsia. Preeclampsia was defined using the criteria of gestational hypertension (blood pressure of 140 or 90 mmHg or more, or an increase of 30 mmHg in systolic or 15 mmHg in diastolic blood pressure compared with values obtained before 20 weeks of gestation), proteinuria (>300 mg/24 h urine protein or greater than 1+ on a dipstick), hyperuricemia (>5.5 mM), and the reversal of hypertension and proteinuria after pregnancy. There was no significant difference in plasma folic acid between the two *MTHFR* genotypes (18± 8.2 ng/mL in CC versus 18.3 ± 5.7 in the TT group). However, maternal plasma folic acid was significantly higher in the preeclamptic (21.1 ± 6.2 ng/mL) than in normal pregnant women (15.5 ± 6.8 ng/mL), despite increased homocysteine concentrations in the women with preeclampsia. Blood pressure did not differ significantly between the CC (systolic and diastolic blood pressure: 136.9 ± 23.9 and 79.7 ± 14.9 mmHg) and TT (129.8 ± 19.6 and 79.8 ± 13.9 mmHg) genotype groups. However, the blood pressure of preeclamptic pregnant women was higher (154.9 ± 12.8 and 91.9 ± 8.5 mmHg) compared to the control group (115 ± 7.9 and 69.3 ± 9.0 mmHg) [64].

Also-Rallo et al. investigated polymorphisms, maternal folate levels, and homocysteine levels in Spanish preeclamptic pregnancies. Preeclampsia was diagnosed in the presence of blood pressure higher than 140/90 mmHg on at least two occasions more than 6 h apart, and proteinuria higher than 300 mg/24 h, always after the 20th week of pregnancy. It included 43 patients with preeclampsia and 122 controls without pregnancy complications. Folate concentrations were significantly higher in the preeclamptic group (18.9 nM) compared with the control group (14.8 nM). They examined two *MTHFR* polymorphisms, C677T and A1298C. The allele distributions were virtually identical between the two groups, and no significant differences were found in the genotype frequencies [65].

Rajkovic et al. investigated the *MTHFR* C677T polymorphism and maternal serum folate concentrations in a Zimbabwean population, including preeclamptic (n = 171) and normotensive control (n = 185) groups. Preeclampsia was defined as a sustained 15 mmHg diastolic increase or a 30 mmHg rise in systolic blood pressure. If first trimester blood pressures were unknown, preeclampsia was defined as persistent blood pressure of at least 140/90 mmHg. Proteinuria was defined as a urine protein concentration of 0.1 g/L or more in at least two random specimens collected at least 4 h apart. They found only one case of the TT genotype (preeclamptic), and the prevalence of the CC and CT genotypes did not differ significantly between the two groups. Folate concentrations were as follows: CC genotype 9.9 ± 5.7 nmol/L (preeclamptic) and 13.6 ± 7.4 nmol/L (control), and CT genotype 8.4 ± 3.4 nmol/L (preeclamptic) and 11.4 ± 6.5 nmol/L (control). Folate levels were significantly lower in the preeclamptic group compared to the control group, but no significant differences were observed between the genotypes [66].

### 3.3. Vitamin D Receptor and Vitamin D

Since the discovery of the *VDR* gene, common SNPs have been specified in the gene. The *VDR* gene has four precise described di-allelic polymorphisms: BsmI (A > G, rs1544410) and ApaI (A > C, rs7975232) on the last intron, and FokI (C > T, rs10735810) and TaqI (T > C, rs731236) polymorphisms lie on the coding exons [67].

Aziz et al. included pregnant Pakistani women in their study (n = 40 in both the control and preeclampsia groups) [68]. They investigated the *VDR* rs7975232 polymorphism and serum vitamin D levels. No associations were found between the polymorphisms and maternal vitamin D levels. It is noteworthy that both groups had very low vitamin D concentrations.

In their 2019 publication, Rezavand et al. included 100 control and 100 preeclamptic (36 severe and 68 mild PE) individuals of Iranian Kurdish ethnic background in their study. Vitamin D was considered adequate when vitamin D levels were above 20 ng/mL. A level between 12 and 20 ng/mL was considered insufficient. A significantly higher percentage of preeclamptic patients (75%) had an insufficient level of vitamin D compared with controls (53.7%). The severity of preeclampsia was not related to the vitamin D level. They investigated the *VDR* FokI, TaqI, and BmsI polymorphisms. The frequency of the Fok1 C allele in preeclamptic patients was significantly higher than those in controls and was associated with a 1.72-fold increased risk of preeclampsia. Neither *VDR* Taq1 nor *VDR* BmsI was associated with the risk of preeclampsia [69]. The authors did not report data on the direct relationships between genotype, vitamin D levels, and blood pressure.

I have already written about Ghorbani and her work group’s study (BMI and vitamin D) [37]. The GT genotype was significantly higher among the preeclamptic group (62%) compared to controls (46%). In the recessive model, the level of vitamin D was significantly lower (17.5 ± 4.2 ng/mL) in the presence of *VDR* rs7975232 GT + GG than the TT genotype (18.9 ± 4.4 ng/mL).

Fondjo et al. investigated preeclampsia cases in Ghana. Preeclampsia was defined according to the ISSHP guidelines. The study included 62 preeclamptic and 100 normotensive control participants. Vitamin D concentrations were significantly lower in the preeclamptic group. Two polymorphisms were detected, rs228570 (Fok1) and rs1544410 (Bsm1). There was no association between the distribution of rs228570 genotypes and preeclampsia. The *VDR* rs1544410 bb genotype reduced the risk of preeclampsia [70].

### 3.4. Proposed New Analysis

The original data attached to the publication were re-analyzed. Serum vitamin D concentrations (ng/mL) were available. Blood pressure values associated with the definition of preeclampsia were provided as specific numbers rather than binary indicators (yes/no), so I treated this as a continuous variable. Blood pressure was plotted against serum vitamin D concentrations using separate scatter plots for each genotype. Control and preeclamptic cases were combined for this analysis. This was necessary because if blood pressure varies depending on vitamin D levels and genotype, individuals with a genotype indicating high risk might be classified as controls (≤140/90 mmHg blood pressure) due to high vitamin D levels mitigating the risk.

**Figure 3:**
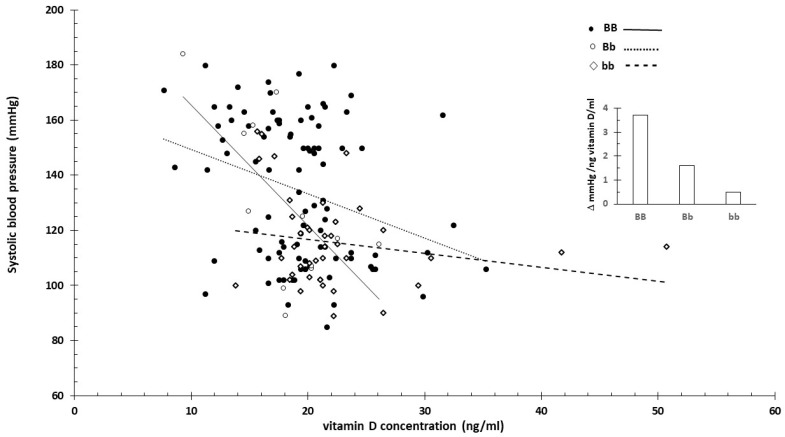
Serum vitamin D concentration and systolic blood pressure by vitamin D receptor B/b genotype. The insert shows the linearized slopes of the data (mmHg/ng vitamin D × mL⁻¹) by genotype. Genotypes BB: _°_, _−−−_; Bb: •, ⋅⋅⋅⋅⋅⋅⋅; bb: ◊, - - - -.

**Figure 4:**
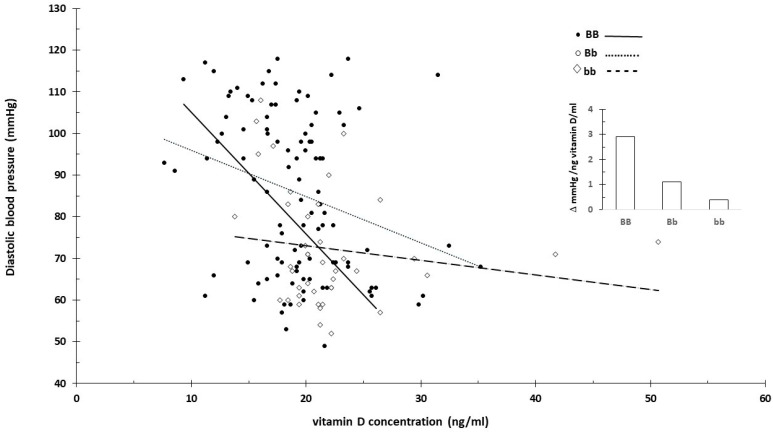
Serum vitamin D concentration and diastolic blood pressure by vitamin D receptor B/b genotype. The insert shows the linearized slopes of the data (mmHg/ng vitamin D × mL⁻¹) by genotype. Genotypes BB: _°_, _−−−_; Bb: •, ⋅⋅⋅⋅⋅⋅⋅; bb: ◊, - - - -.

For carriers of the BB (B allele) genotype, blood pressure significantly decreased with the increasing vitamin D concentration (Figure 3 and Figure 4). In contrast, the effect was minimal for the bb genotype.

**Figure 5:**
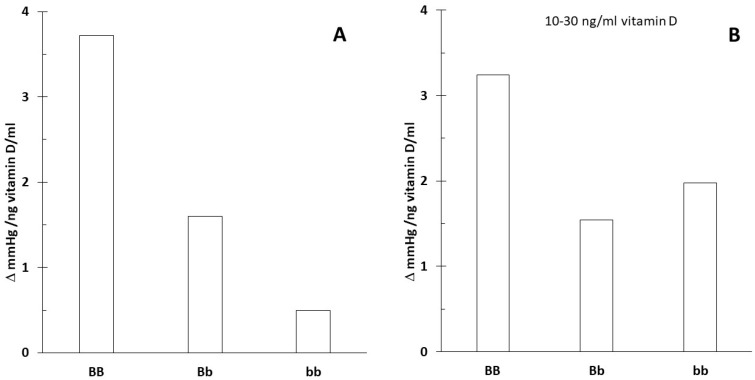
Changes in systolic blood pressure at different vitamin D concentrations in individuals with the vitamin D receptor B/b genotype. The figures display the slope of linearized data (mmHg/ng vitamin D × mL⁻¹). (**A**) shows the slopes calculated from all data (7.6–50.7 ng/mL vitamin D), while (**B**) presents the slopes derived from data within the 10–30 ng/mL vitamin D concentration range, categorized by genotype.

This was likely related to the higher vitamin D levels associated with the bb genotype. When excluding extreme vitamin D values that might distort the effect, the impact associated with the genotypes diminished. Figure 5 shows the slope of systolic blood pressure across all data (Figure 5A) and specifically between 10 and 30 ng/mL vitamin D concentrations (Figure 5B).

**Figure 6:**
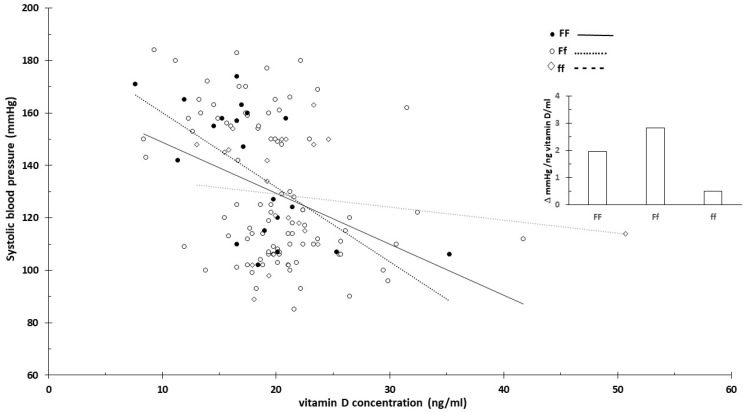
Serum vitamin D concentration and systolic blood pressure by vitamin D receptor F/f genotype. The insert shows the linearized slopes of the data (mmHg/ng vitamin D × mL⁻¹) by genotype. Genotypes FF: _°_, _−−−_; Ff: •, ⋅⋅⋅⋅⋅⋅⋅; ff: ◊, - - - -.

**Figure 7:**
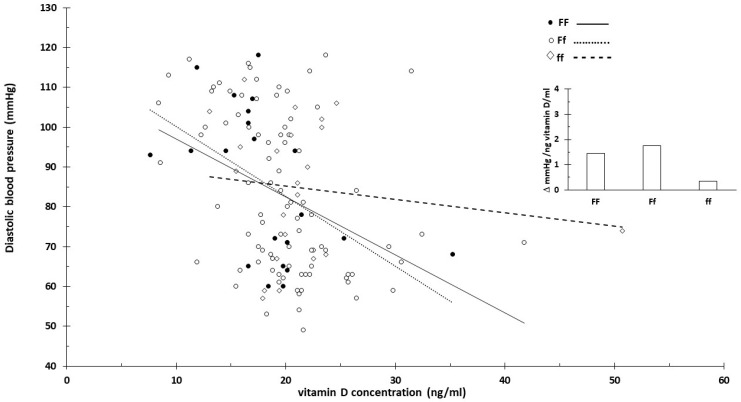
Serum vitamin D concentration and diastolic blood pressure by vitamin D receptor F/f genotype. The insert shows the linearized slopes of the data (mmHg/ng vitamin D × mL⁻¹) by genotype. Genotypes FF: _°, −−−_; Ff: •, ⋅⋅⋅⋅⋅⋅⋅; ff: ◊, - - - -.

No clear vitamin D concentration-dependent blood pressure changes were observed for the F/f genotype (Figure 6 and Figure 7).

**Figure 8:**
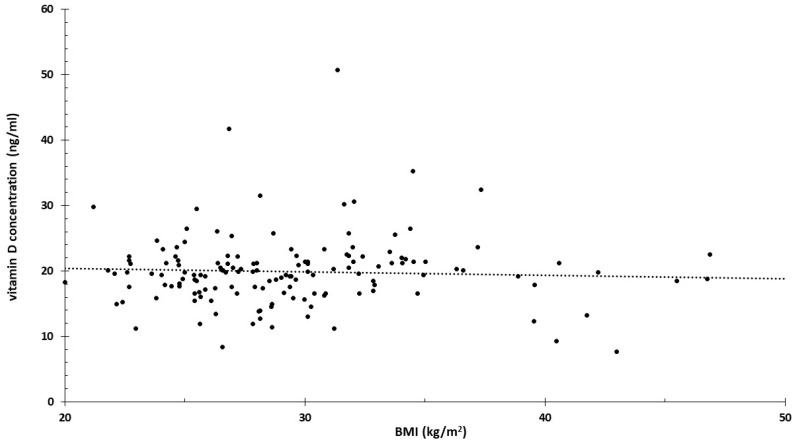
Participants’ vitamin D levels and BMI values.

It is well known that obesity is associated with vitamin D deficiency. A very strong correlation was found between the increase in BMI and the decrease in vitamin D levels. According to the study, for every 1 kg/m² increase in BMI, the 25-hydroxyvitamin D level decreased by 1.15% [71]. This also implies that the presented results may be distorted by BMI. The data necessary for the calculation were derived from a population in which serum vitamin D levels were practically independent of BMI (Figure 8). Figure 8 shows the data of all participants (controls and patients) involved in the study. The minimal decrease calculated from the linear regression was one-fifth of the −1.15% mentioned in the study.

### 3.5. SIRT 1 Polymorphisms and Levels of Micronutrients

Sirtuin-1 (*SIRT1*) is a member of the sirtuin protein family, comprising seven proteins (SIRT1–7) in humans, and functions as a class III histone deacetylase that is dependent on NAD^+^. *SIRT1* plays a crucial role in modulating oxidative/antioxidative signaling pathways and regulates a broad spectrum of genes involved in antioxidant defense mechanisms, including those encoding glutathione peroxidase, superoxide dismutase (SOD), and catalase. Research indicates that certain single-nucleotide polymorphisms (SNPs) within the *SIRT1* gene are correlated with variations in blood pressure [72]. Specifically, three SNPs have been identified: rs7895833 A > G in the promoter region, rs7069102 C > G in intron 4, and rs2273773 C > T, a silent mutation in exon 5 [73].

Summarizing the genotypes associated with micronutrients, Table 2 shows the results.

## 4. Discussion

Nutrigenetics is a new scientific field, and research in this area began in the 21st century [74].

### 4.1. Obesity

Obesity is one of the high-risk factors for preeclampsia. By pairing genotype with BMI, new risk factors can be identified. I found several examples of BMI-dependent genetic risk. One such example is the *AVP* rs3729965 polymorphism. The T allele (CT genotype) increases the risk of preeclampsia in obese pregnant women [38]. Two studies reached similar conclusions when investigating the renin rs5707 polymorphism. The protective effect of the AA polymorphism is only significant when the BMI is less than 24 [19,22]. The *AGTR2* C4599A A polymorphism is a risk factor for preeclampsia only when the BMI exceeds 25 [25]. Interestingly, this polymorphism increases the risk of hypertension only in women [75].

### 4.2. Micronutrients

The main finding of this study was that the use of scatter plot analysis may enable more effective nutritional counseling for the prevention of preeclampsia based on genotype knowledge. For individuals with the *VDR* BB genotype, vitamin D supplementation may be more effective for preventive purposes.

Several studies have highlighted the importance of optimizing vitamin intake according to polymorphisms. For certain alleles, vitamin supplementation can also enhance the effectiveness of pharmacological treatments [76].

Previous studies generally did not find significant differences in folic acid and homocysteine concentrations when samples were grouped according to *MTHFR* genotypes. The folic acid concentration was generally higher in samples with the TT (C677T) genotype.

Folic acid supplementation during pregnancy is widely accepted and routinely administered. Folic acid deficiency in pregnant women increases the risk of birth defects, especially neural tube malformations [77]. Excessive folic acid supplementation can increase the risk of breast cancer in pregnant women, leads to zinc deficiency in the body, causes abnormal fetal development, and covers up vitamin B12 deficiency [78,79,80]. A recent meta-analysis showed that folic acid supplementation during pregnancy could not be proven to reduce the risk of preeclampsia [81]. Folic acid can increase the nitric oxide (NO) levels and restore Type II diabetes-associated endothelial dysfunction [82].

Genome-based nutritional counseling during pregnancy is already in use. Yu et al. examined three polymorphisms affecting folate metabolism (*MTHFR* C677T, *MTHFR* A1298C, and *MTRR* A66G). They used combinations of genotypes to assess risk, forming low-, medium-, and high-risk groups. The low-risk group received 400 µg/day of folic acid supplementation from the third month before conception until the end of pregnancy. In the medium-risk group, the supplementation was increased to 800 µg/day during the first trimester. The high-risk group received 800 µg of folic acid daily both before conception and during the first trimester. The incidence of GDM was significantly lower in the case group than in the control group. Particularly, in high-risk pregnant women, there were fewer cases of GDM [80].

### 4.3. Future Nutrigenetic Studies

The presented studies were generally not conducted with a nutrigenetic purpose. The available studies typically investigated the most common polymorphisms of the examined gene. The results are categorized into two groups (e.g., control–preeclampsia, normal–overweight, and insufficient–sufficient vitamin D/folate levels).

In future nutrigenetic studies related to preeclampsia, the focus will likely continue to be on *MTHFR*. Methylation transfer is influenced by folic acid and vitamins B6 and B12, which can only be obtained through diet. Insufficient function of *MTHFR* increases homocysteine levels. Homocysteine is a specific amino acid, and its formation is linked to this process. More than 900 polymorphisms of *MTHFR* are known [83]. Therefore, MTHFR is ideal for nutrigenetic studies.

### 4.4. Target Gene and Metabolite Selection

When selecting a target gene (protein), it is advisable to consider kinetic/expression properties and, accordingly, measure the nutrient/metabolite.

I present two examples (nitric oxide synthase (NOS) and glutamate oxaloacetate transaminase 1 (GOT1)) that are not included in this publication.

NO effectively lowers blood pressure, and NOS synthesizes it from arginine. The function of NOS requires Ca^2+^ and tetrahydrobiopterin. Inadequate NOS function may play a role in the development of preeclampsia, which can be associated with polymorphisms [84,85,86,87] in endothelial NOS expressed in the placenta [74]. The eNOS Glu298Asp polymorphism reduces eNOS expression [88,89]. Arginine supplementation, particularly with decreased serum arginine levels, may be more effective in individuals with the Asp polymorphism than with the Glu type. Arginine supplementation is effective in pregnant women with preeclampsia even without knowledge of the genotype [90,91,92]. The concentration of tetrahydrobiopterin in the placenta remains around the eNOS K_M_ value throughout pregnancy [93,94,95]. A subset of preeclamptic placental eNOS has reduced BH4 affinity (BH4-insensitive placenta) [93,96]. To reach physiological concentrations, the tetrahydrobiopterin level would need to be increased approximately 100-fold, making it impractical to measure tetrahydrobiopterin or its metabolite concentration. The physiological concentration of BH4-insensitive placental eNOS activity can be increased with pravastatin [80]. This effect is due to pravastatin enhancing placental arginine uptake [97]. Therefore, in BH4-insensitive preeclampsia studies, it is also recommended to determine the arginine concentration.

GOT1 interconverts two non-essential amino acids: glutamate and glutamine. In a patient with preeclampsia, a mutation likely altering enzyme affinity has been identified [89], though this example is hypothetical. When examining nutrients, the B6 concentration should be measured, as it is essential for the enzymes’ function. For metabolites, it is advisable to measure glutamate between the two readily available amino acids (glutamate and glutamine), as determining glutamine is methodologically challenging [98,99,100]. In severe preeclampsia, the blood glutamate concentration significantly increases, and glutamine is absent from amino acid analysis [101].

### 4.5. Data Evaluation

Currently, threshold values are determined for a given population (e.g., vitamin D deficiency is defined as below 20 ng/mL of serum). Data should be analyzed by genotype, pairing relevant measurable values concerning the disease (e.g., AA genotype, arginine concentration, and blood pressure). It is also useful to conduct the analysis on the entire study group, not separating control and patient groups. This approach may help define beneficial genotype-specific thresholds. For example, the incidence of preeclampsia (140/90 mmHg blood pressure) significantly decreases if the AA genotype achieves a metabolite concentration of 10 units, Aa genotype 20 units, and aa genotype 40 units.

## 5. Strengths and Weaknesses

The strength of the current review is that it proposed innovative data analysis not previously used for genome-based nutritional counseling. As illustrated by the presented example (Figure 1, Figure 2, Figure 3, Figure 4 and Figure 5), scatter plot analysis can be beneficial.

Clinical experience confirms that patient compliance improves when the healthcare provider supports recommendations with personalized evidence. For instance, recommending weight loss before conception to an obese woman because she carries a renin mutation that significantly increases the risk of preeclampsia with a high BMI.

The weakness of the publication is the high heterogeneity of the data, as the studies investigated different genes/polymorphisms. The sample sizes were also low. Each genotype was usually analyzed in only one study, so the conclusions may change with subsequent studies. Much more data will be needed for well-founded conclusions and recommendations.

## 6. Conclusions

Nutrigenetics is a relatively new scientific discipline that emerged in the 21st century. Its relevance is particularly apparent in conditions such as preeclampsia, where genetic predispositions, influenced by diet and micronutrient intake, can impact disease risk.

Overweight is a major risk factor for preeclampsia, and genetic risk can be modulated by BMI. For instance, certain polymorphisms, such as *AVP* rs3729965 and *AGTR2* C4599A, exhibit obesity-dependent risks. These findings suggest that pairing genotype with BMI can help identify new risk factors, making a case for personalized interventions in high-BMI individuals.

The study highlighted the importance of adjusting micronutrient intake based on genetic profiles to prevent preeclampsia. Vitamin D and folic acid are of particular importance. However, while folic acid supplementation is routine, excessive intake poses risks and does not conclusively reduce preeclampsia risk. Emerging genome-based counseling approaches that tailor folate supplementation based on polymorphisms of *MTHFR* could show positive outcomes in reducing preeclampsia risk.

In summary, integrating genetic data with personalized nutrition, particularly concerning BMI and micronutrient intake, offers a promising strategy for preventing or mitigating preeclampsia. Establishing genotype-specific thresholds for beneficial metabolite concentrations could optimize personalized nutritional interventions. However, more targeted nutrigenetic studies are necessary to refine these approaches.

## Figures and Tables

**Table 1:** Effect of BMI-specific polymorphisms on preeclampsia.

Reference	Polymorphism	Gene	Region	Effect of Allele	BMI
Socol (2024) [26]	rs5707	renin (*REN*)	intron	protective: AA	BMI < 24 *
Yu (2019) [30]	rs5707	renin (*REN*)	intron	risk: AC	BMI ≥ 24 *
He (2023) [31]	rs7079	angiotensinogen (*AGT*)	3′UTR side	risk: TT	BMI < 25 *
Amosco (2019) [39]	rs1014064	*ACVR2*	intron	risk: G	BMI ≥ 25.1 *
Madar-Shapiro (2018) [40]	rs3764843	*LGALS13*	promoter	risk: AA	BMI ≥ 35 *
Karppanen (2016) [36]	rs4606	*RGS2*	3′UTR side	risk: GG/CG	25 ≤ BMI < 30 *
Zhou (2013) [33]	C4599A	*AGTR2*	3′UTR side	risk: A	BMI ≥ 25 * BMI < 25 N.E.
Zhang (2005) [41]	rs4994	*ADRB3*	Trp64Arg	protective: AA	BMI < 30 *
Enquobahrie (2005) [45]	rs1800588	*LIPC*	promoter	risk: TT	BMI ≥ 25 * BMI < 25 N.E
Erfanian (2019) [38]	rs3729965	*AVP* (arginine vasopressin)	promoter	risk: T	BMI ≥ 30 *
Huang (2022) [35]	rs2106809	*ACE2*	intron	risk: AA	BMI ≥ 23 *
Abramova (2022) [50]	rs805303	*BAG6*	intron	protective: A	BMI ≥ 25 * BMI < 25 N.E.
Abramova (2022) [50]	rs167479	*RGL3*	Pro162His	risk: G	BMI ≥ 25 * BMI < 25 N.E.

BMI: body mass index (kg/m^2^); N.D.: no data; N.E.: no effect; *: there is an association between preeclampsia and genotype.

**Table 2:** Associations between micronutrients and polymorphisms.

Reference	Polymorphism	Gene	Region	Effect of Allele
Mislanova (2011) [62]	rs1801133	MTHFR	Ala222Val	C/T: lower folate concentration
Williams (2004) [63]	rs1801133	MTHFR	Ala222Val	polymorphism is not an important risk marker
Powers (2003) [64]	rs1801133	MTHFR	Ala222Val	no difference in folate concentration between the alleles
Also-Rallo (2005) [65]	rs1801133	MTHFR	Ala222Val	alleles have no effect
Also-Rallo (2005) [65]	rs1801133	MTHFR	Glu429Ala	alleles have no effect
Rajkovic (2000) [66]	rs1801133	MTHFR	Ala222Val	no difference in folate concentration between the alleles
Aziz (2023) [68]	rs7975232	VDR	intron	alleles have no effect
Rezavand (2019) [69]	rs10735810 rs1544410 rs731236	VDR	exon intron exon	no correlation was investigated
Ghorbani (2021) [37]	rs7975232	VDR	intron	vitamin D concentrations lower in GT/GG than the TT genotype
Fondjo (2024) [70]	rs228570 rs1544410	VDR	intron intron	no vitamin D concentration test according to genotype
Khadir (2024) [73]	rs7895833 rs7069102 rs2273773	SIRT1	promoter intron exon	zinc decreasing AA GG TT
Khadir (2024) [73]	rs7069102	SIRT1	intron	Cu level increased: TT

MTHFR: methylenetetrahydrofolate reductase; VDR: vitamin D receptor; SIRT1: sirtuin 1.
